# P02-011 - TRAPS syndrome debuted as systemic JIA

**DOI:** 10.1186/1546-0096-11-S1-A118

**Published:** 2013-11-08

**Authors:** T Sleptsova, E Alexeeva, E Mitenko, S Valieva, T Bzarova, R Denisova, K Isaeva

**Affiliations:** 1 Department of Rheumatology, Scientific Centre of Children’s Health, Moscow, Russian Federation

## Introduction

Tumor necrosis factor receptor-associated periodic syndrome (TRAPS) is characterized by periodic fever, cutaneous rash, conjunctivitis, lymphadenopathy, abdominal pain, myalgia, and arthralgia. It is a rare autosomal dominant disease and strongly associated with heterozygous mutations in the tumor necrosis factor (TNF) receptor super family 1A (TNFRSF1A) gene. The great diversity of manifestations and the difficulties in genetic analyses make the diagnosing of this disease a challenge. Our aim was to report on case of autoinflammatory syndromes that is considered to be rare entity.

## Case report

A 16-year-old Caucasian boy presented at the age of 15 years with fever (39 ºC), weakness, lymphadenopathy, splenomegaly and arthralgia. The laboratory tests revealed anemia, leukocytosis, thrombocytosis, ESR of 80 mm, CRP of 90 mg/l (normal < 0.5). A diagnosis of systemic juvenile idiopathic arthritis (JIA) was made and the patient was treated with corticosteroids, NAIDs and gamma globulin. Over the next 6 months, he presented skin rash regarded as reaction to drugs. No abdominal pain or conjunctivitis was noted. Pulse therapy with methylprednisolone and constant administration NAIDs were needed to control fever and pain. After extensive work-up of infectious etiology, an oncological disease with negative results he underwent diagnostic laparoscopic surgery for lymph node biopsy because of abdominal lymphadenopathy, without findings. Questioned further autoinflammatory syndrome was suspected. DNA analysis showed a mutation present in exons 9 of the TNFRSF1A gene (deletion c.792delT), thus resulting in a diagnosis of TRAPS. He had partial clinical response to corticosteroids. However, the treatment response to TNF-α inhibitor infliximab was dramatic. At the present he still presents rash but no fever and lymphadenopathy.

## Discussion

Here, we report a patient with TRAPS who recovered from steroid dependency by infliximab and kept remission with infliximab. The patient was thought to have had systemic JIA. Failure to respond to therapy, good response only to very high doses of corticosteroids led us to suspect autoinflammatory syndrome. Clinical features are important for the diagnosis, but confirmation is obtained through genetic analysis. Autoinflammatory syndromes should be considered in patients with fever of unknown origin and the clinicians must be aware of the diversity of manifestations and diagnostics of these conditions.

## Disclosure of interest

None declared.
